# Dietary Energy Density Is Associated with Biomarkers of Chronic Diseases—A Cross-Sectional Study of School-Aged Children in Rural Mexico

**DOI:** 10.1016/j.cdnut.2024.102096

**Published:** 2024-02-17

**Authors:** Gerardo A Zavala, Olga P García, Dolores Ronquillo, Colleen M Doak, Maria del Carmen Caamaño, Mariela Camacho, Jorge L Rosado

**Affiliations:** 1 Department of Health Sciences, University of York, Heslington, York, United Kingdom; 2 School of Natural Sciences, Universidad Autónoma de Querétaro, Santiago de Querétaro, Mexico; 3 Public Health, St Ambrose University, Davenport, IA, United States

**Keywords:** dietary energy density, obesity, inflammation, insulin resistance, children

## Abstract

**Background:**

Dietary energy density (DED) is associated with chronic disease markers in adults. However, results in children are still controversial.

**Objective:**

To evaluate the DED of children and its association with obesity and biomarkers of chronic disease.

**Methods:**

In this cross-sectional study, we recruited 284 children (6–10 y) from rural Mexico. Dietary intake was assessed using three 24-h recalls. DED was calculated for “foods only” (DED_fo_) and for “foods and beverages” (DED_fb_). Weight, height, and body fat percent (dual-energy X-ray absorptiometry) were measured. Inflammatory cytokines, lipid profile, leptin, and insulin resistance were determined from a fasting blood sample.

**Results:**

DED_fo_ was 1.91 ± 0.36 kcal/g and DED_fb_ was 1.36 ± 0.31 kcal/g. Higher DED_fo_ and DED_fb_ were associated with higher risk to have insulin resistance [odds ratio (OR) = 3.92, 95% confidence interval (CI): 1.66, 9.22, *P* < 0.01; OR = 3.51, 95% CI: 1.25, 9.87, *P* = 0.02, respectively]. Higher DED_fo_ was associated with higher risk of higher leptin levels (OR = 3.17, 95% CI: 1.01, 10.23). Also, DED_fo_ and DED_fb_ were associated with higher concentrations of cholesterol (*β* = 11.67, 95% CI: 1.81, 19.53, *P* = 0.03; and *β* = 11.74, 95% CI: 2.69, 20.74 *P* = 0.01, respectively) and higher odds of having high insulin concentrations (OR = 2.52, 95% CI: 1.26, 5.06, *P* = 0.01; and OR = 2.95, 95% CI: 1.30, 6.70, *P* = 0.01). DED_fo_ and DED_fb_ were not associated with any measure of obesity and inflammatory cytokines in the adjusted models.

**Conclusions:**

DED was associated with higher leptin and cholesterol concentrations, and having insulin resistance, but not with any measure of obesity or inflammation. Reducing DED may reduce risk of cardiovascular disease and improve insulin sensitivity in school-aged children.

## Introduction

The combined prevalence of overweight and obesity in Mexican school-aged children is 37.3%, and these children have an increased risk of impaired lipid profile, oxidative stress, inflammation, and insulin resistance [1]. Up to 50% of Mexican school-aged children with obesity have insulin resistance, >60% have high triglycerides concentration, and 50% have high cholesterol levels [[2], [3], [4]].

The dietary energy density (DED), defined as the amount of energy per weight unit of food (that is, kcal/g) [5], has been identified by the WHO as a major contributor to childhood obesity highlighting the importance of reducing intake of high energy-dense foods [6,7]. Energy-dense foods often contribute to excessive caloric intake, leading to weight gain and obesity, and contribute to elevated levels of blood glucose, insulin, and triglycerides [8].

Lowering the energy density of food lowers energy intake, which, if sustained over time, may result in an effective weight loss strategy compared with calorie restriction [9]. A systematic review, evaluating 17 studies in adults, reported that a reduced DED was associated with weight loss [10]. According to the same review, the information concerning the association between DED and obesity in children is scarce, and the few available studies have shown conflicting results. Some studies have found a positive association between DED and childhood obesity [11,12], whereas other studies have found an association only with some predictors of obesity (that is, higher DED associated with lower household incomes and enrollment in the food stamp program) [13] or no association at all [10,14]. The lack of a significant association between DED and obesity in children has been suggested to be attributed to individual variations in metabolism and the complex interplay of genetic and environmental influences on childhood weight status [10,14].

There is also limited information regarding the relationship of DED with metabolic markers in children. Also, in children living in rural communities of low- and middle-income countries, where the rates of overweight and obesity are increasing rapidly, the DED and its relationship with obesity is not known [15]. Particularly in rural Mexico it is common for children to eat energy-dense foods such as “*atole*” (maize-based sweetened hot beverage), “*tamales*” (seasoned meat, lard, and maize flour steamed dish), deep fried tacos, “*churros*” (wheat flour dough, fried in vegetable oil, and covered in sugar), home-made fried crisps, and sweet pastries, and also vegetables are commonly consumed fried as part of cooked dishes [16]. In addition, the availability and affordability of highly processed foods in these communities may increase the DED in populations living in rural settings [16].

DED can be calculated using “only food” excluding all beverages or using “food and beverages” that can include either all beverages or only caloric beverages [10,17]. Little is known about the best method to assess DED in different populations, such as different age ranges, ethnicities, and different nationalities. Therefore, to measure DED, it is recommended to use both, “food only” and “food and beverages” [17].

Given the prevalence of overweight and obesity and the rising prevalence of metabolic syndrome in Mexican school-age children [18,19], and the possible contribution of DED to this public health problem, it is important to evaluate DED in children from rural areas in Mexico and determine its associations with obesity and biomarkers of chronic disease. Thus, among school-age children living in rural Mexico, the objectives of this study are *1*) to assess the DED; *2*) to assess the association between DED and measurements of obesity; and *3*) to assess the association of DED with biomarkers of chronic disease.

## Methods

### Subjects and study design

In this cross-sectional study, a total of 293 school-aged children between 6 and 10 y of age were recruited from 2 rural communities: Santa Cruz and Santa María Begoña. These communities are 3 km away from each other and both belong to the municipality of El Marques in the state of Querétaro, México. The children were recruited from the elementary schools of Dieciseis De Septiembre, in the community of Santa Cruz, and Benito Juarez in the community of Santa María Begoña. These communities have only one elementary school each, and all the school-aged children attending were invited to participate in the study. The ethnicity is predominantly mestizo (mixed-race individual), and agriculture is the main economic activity. The nearest metropolitan center is the city of Querétaro, which is 35 km away from the communities. In this region, the diet includes staple foods such as beans and corn-tortilla. The main animal food sources are eggs, dairy products, pork, and chicken. Seasonal fruits and vegetables are commonly consumed. Diets in these populations also include high energy-dense foods with low nutritional quality such as desserts and pastries, cookies, fried chips, “*churros*,” and chocolate.

We calculated that a sample size of 168 was needed to detect statistical difference of 0.70 score points between lower and higher age-adjusted BMI groups with an 80% of power in DED, assuming a pooled SD of 0.23 [20].

Parents of recruited children received oral and written information about the study and signed an informed consent. Children were excluded from the study if they had any mental or physical disability, if they were previously diagnosed with diabetes, or if they were following a special dietary regimen. The study protocol was approved by the Bioethics Committee of the Universidad Autónoma de Querétaro (UAQ) and followed the Declaration of Helsinki guidelines.

### Biomarkers of chronic disease

A fasting blood sample (7 mL) was collected by a trained laboratory technician at the community’s local clinic. Children were instructed not to eat anything ≥12 h before the blood sample was collected early in the morning. Plasma and serum were separated in blood samples by centrifugation at 400 × *g* for 15 min (Beckman Allegra 21R), and aliquots were stored at −70°C for later analysis. All biochemical analyses were performed in duplicate in the Human Nutrition Laboratory at UAQ.

Serum insulin concentration was determined by a commercial ELISA kit (Bio Quant) using a microplate photometer (Multiskan Ascent; Thermo Electron Corporation). Fasting glucose was measured in plasma by a colorimetric/enzymatic method using a commercial kit (Glucose Elitech) and a clinical analyzer (Bayer RA-50; Bayer Diagnostics), and concentrations of 100 mg/dL and above were considered as high. Insulin resistance was determined using the HOMA with the following formula: HOMA = insulin (μU/m) × glucose (mmol/L)/22.5. Insulin resistance was defined with a HOMA value >3.16 [21]. Triglycerides and total cholesterol were determined in plasma using commercially available kits (Cholesterol; Elitech; Triglycerides; Elitech) using a clinical chemical analyzer (Bayer RA-50; Bayer Diagnostics). High triglycerides were defined when concentration was >110 mg/dL and high cholesterol when concentration was >200 mg/dL. Plasma HDL cholesterol and LDL cholesterol were measured by spectrophotometry (Genesis 20 ThermoSpectronic; Thermo Electron Corp) using commercially available kits (Cholesterol HDL; Elitech; Cholesterol LDL; Spinreact).

Commercial ELISA kits were used to determine IL-6 (Millipore), TNF-α (Millipore), high-sensitivity C-reactive protein (CRP) (Bioquant), and leptin concentrations (Millipore). The ELISAs were read using a microplate photometer (Multiskan Ascent; Thermo Electron Corporation).

### Anthropometry

Children’s weight, height and waist circumference were measured by trained and standardized personnel at the Nutrition Clinic at UAQ. All measurements were done in duplicates following WHO procedures [22]. Weight was measured using a digital scale (SECA mod 813), height using a stadiometer (SECA mod 206), and waist circumference was determined using a flexible glass anthropometric tape (SECA mod 813). On the basis of the WHO criteria for children 5–19 y, BMI-for-age *z*-score (BMIz) was calculated using the Anthroplus software (Geneva: WHO, 2009). Underweight was defined as 2 *z*-scores below the WHO reference median, overweight as 1 SD above the WHO reference median and obese as 2 SDs above the reference median of the BMIz [23].

### Body composition

A certified technician measured total body fat percent in the children using dual-energy X-ray absorptiometry (Hologic Mod Explorer). Abdominal and total fat content (kg) were estimated following procedures previously described by Hill et al. [24]. High body fat was considered above 30% for girls and above 25% for boys [25].

### Diet and dietary energy density

Trained nutritionists applied three 24-h recalls (24hR) on 3 separate days (2 weekdays and 1 weekend) to the caregiver in the presence of their child. Dietary macronutrient composition (that is, carbohydrates, fats, protein, and fiber) and daily caloric intake (kcal) were calculated from the average of the three 24hR using food composition tables from the National Institute of Medical Science and Nutrition “Salvador Zubiran” [26] in Mexico and the USDA Nutrient Database.

DED was calculated using both, the “food only” and the “foods and beverages” methods; outliers (that is, implausible values) <3 SD were removed from the study according to standard procedures [17,27]. For the DED of food only (DED_fo_), the caloric content (kcal) of all the foods was obtained from the 24 h, and divided by the mean of the total weight (g) of the foods (DED_fo_ = kcal/g of food only). For the DED of foods and caloric beverages (DED_fb_), the caloric content (kcal) of all the foods and caloric beverages was divided by the total weight (g) of the foods and caloric beverages (DED_fb_ = kcal/g of food and beverage).

### Covariables

A series of variables that are known to influence dietary intake and obesity were collected. Physical activity (PA) was estimated by a validated questionnaire [28]. The children’s caretakers were asked to report the amount (h/d) of moderate and intense physical activities, according to the compendium of physical activities of Ainsworth [29]. Educational level of the caregiver was recorded in years of formal education.

### Statistical analysis

All statistical analyses were performed using the statistical package SPSS, version 20.0 (IBM). A descriptive analysis of all the variables was conducted and results are presented as means ± SDs and percentages for categorical variables. Comparisons between all the measured variables between children with normal and high percentage of body fat were assessed by the chi-square test or *t*-test for independent samples. The distribution of dependent variables (BMI categories, waist circumference, percentage of body and abdominal fat, and biomarkers of chronic disease) was explored to confirm a normal distribution with the Kolmogorov–Smirnov test. Linear regression models were performed for variables that were normally distributed to assess the association between DED_fo_ and DED_fb_ (as independent variables) with different body fat measurements (as dependent variables) controlling for age, sex, and level of education of the caregiver (as possible confounders). We used logistic regression analyses to assess the association between the dependent variables that were not normally distributed (IL-6, TNF-α, CRP, leptin, insulin, and HOMA) and measures of body fat controlling for fat percent, age, sex, caregiver’s education level, and PA (as possible confounders). CRP was also added as a confounder for the models of IL-6, TNF-α, leptin, insulin, and HOMA. The values of the dependent variables were categorized into 2 levels: below and above the median for leptin (0.36 mg/L), IL-6 (1.86 pg/mL), TNF-α (3.5 pg/mL), and insulin (10.85 U/mL), and we used the cutoff point for high risk of systemic inflammation for CRP (>2.99 mg/L) [30] and for HOMA below and above the cutoff point of 3.16 [21].

## Results

From the 293 children included in the study, 5 did not provide a blood sample and 4 reported an unplausible DED_fo_ or DED_fb_ value (<3 SD). Fifty-two percent of the children had high body fat; of those, 31.1% were females. As shown in Table 1, 53% of the children had a high percentage of body fat (>25% for girls and >30% for boys), and according to their BMI, 18% were classified as overweight, 9% as obese, and only 2% were underweight. Compared with children with normal fat, children with elevated levels of body fat had significantly higher concentrations of triglycerides (101.73 mg/dL, SD 45.31 compared with 75.69 mg/dL, SD 30.66; *P* < 0.001) and total cholesterol (152.31 mg/dL, SD 22.46 compared with 144.41 mg/dL, SD 23.81; *P* = 0.032). Specifically, 18.7% of the children with high body fat had high triglyceride levels, whereas only 4.5% of those with normal body fat had high triglyceride concentrations. In addition, 6% of children with normal body fat and 13% of those with high body fat had insulin resistance, as indicated by a HOMA >3.16.

**TABLE 1:** Main characteristics of the studied children according to their body fat content

	Normal body fat (*n* = 131) Mean ± SD or Percentage (*n*)			High body fat (*n* = 153) Mean ± SD or Percentage (*n*)			Overall (*n* = 284) Mean ± SD or Percentage (*n*)			*P*
Age (y)	7.81	±	1.56	8.16	±	1.55	7.99	±	1.56	0.053
Weight (kg)	23.61	±	4.77	31.16	±	9.05	27.66	±	8.28	<0.001
Height (cm)	123.93	±	9.18	128.30	±	10.15	126.27	±	9.94	<0.001
Waist circumference (cm)	54.50	±	5.04	64.59	±	9.42	59.91	±	9.20	<0.001
Height-for-age (*z*-score)	−0.85	±	0.97	−0.37	±	0.93	−0.59	±	0.98	<0.001
BMI-for-age (*z*-score)	−0.44	±	1.00	1.01	±	1.07	0.34	±	1.26	<0.001
Body fat (%)	23.34	±	3.47	33.89	±	4.87	29.00	±	6.79	<0.001
Abdominal fat (%)	20.15	±	4.15	34.69	±	7.73	27.95	±	9.62	<0.001
Caregiver’s education (y)	4.24	±	1.58	4.07	±	1.27	4.15	±	1.43	0.081
Moderate of intense physical activity (h/d)	1.84	±	1.23	1.66	±	1.34	1.72	±	1.27	0.126
Triglycerides (mg/dL)	75.69	±	30.66	101.73	±	45.31	89.66	±	41.25	<0.001
High triglycerides (%)	4.5		(13)	18.7		(53)	23.2		(66)	0.002
Total cholesterol (mg/dL)	144.41	±	23.81	152.31	±	22.46	148.65	±	23.39	0.032
High total cholesterol (%)	5.6		(16)	11.3		(32)	17		(48)	0.103
HDL (mg/dL)	49.55	±	7.95	47.06	±	8.52	48.22	±	8.36	0.234
LDL (mg/dL)	80.35	±	17.97	85.38	±	83.05	48.22	±	8.36	0.076
Leptin (ng/mL)	1.40	±	0.83	5.92	±	5.90	3.83	±	4.91	N/A
Glucose (mg/dL)	82.03	±	6.87	83.31	±	7.25	82.49	±	7.07	0.343
High fasting glucose (%)	0.4		(1)	1.4		(4)	1.8		(5)	0.043
Insulin (uU/mL)	11.15	±	5.59	13.48	±	8.18	12.40	±	7.40	0.093
HOMA (%)	4.2		(12)	14.8		(42)	19.0		(54)	<0.001
CRP (mg/L)	0.98	±	1.35	0.96	±	1.73	0.97	±	1.53	N/A
CRP (%)	5.2		(15)	13.7		(39)	18.9		54	<0.001
IL-6 (pg/mL)	1.93	±	1.13	4.16	±	5.06	3.14	±	3.92	N/A
TNF-α (pg/mL)	3.29	±	1.88	5.04	±	3.35	4.23	±	2.87	N/A

Normal body fat, <25% for girls and <30% for boys; high triglycerides, >110 mg/dL; high total cholesterol, <170 mg/dL; high fasting glucose, >100 mg/dL; HOMA, >3.16; CRP%, >2.99 mg/L; *P* value comes from *t*-test for continuous variables and chi-squared for categorical variables.

Abbreviations: CRP, C-reactive protein; N/A, variables were not normally distributed and the *t*-test was not performed.

There was low variability among the population studied in meal frequency. Most of the children have 4 meals at very similar times: “*desayuno*” before school (between 7 and 8 am), “*almuerzo*” at school (approximately at 12 pm), “*comida*” just after school (between 2 and 3 pm after school), and “*cena*” (between 6 and 7 before bed).

The mean of the DED_fo_ for all the sample was 1.91 kcal/g (SD = 0.36) for the “food only” method and 1.36 kcal/g (SD 0.31) for the “food and beverages” method (Table 2). Children meeting the recommended daily allowance (RDA) of caloric intake of >1600 kcal/d reported a higher DED_fo_ of 1.95 (SD = 0.29) compared with those not meeting the RDA, who had a DED_fo_ of 1.85 (SD = 0.35), with a significant difference (*P* = 0.011). Similarly, children meeting the caloric RDA also had a higher DED_fb_ of 1.41 (SD = 0.27) in comparison with those not meeting the RDA, who showed a DED_fb_ of 1.30 (SD = 0.28) (*P* = 0.034).

**TABLE 2:** Dietary energy density and macronutrients of the studied children according to their body fat content

	Normal body fat (*n* = 131) Mean ± SD			High body fat (*n* = 153) Mean ± SD			Overall (*n* = 284) Mean ± SD			*P*
DED_fo_ (kcal/g)	1.85	±	0.32	1.95	±	0.34	1.91	±	0.36	0.011
DED_fo_ (kJ/g)	7.74	±	1.33	8.16	±	1.42	7.99	±	1.50	0.011
DED_fb_ (kcal/g)	1.35	±	0.30	1.37	±	0.28	1.36	±	0.31	0.611
DED_fb_ (kJ/g)	5.64	±	1.25	5.73	±	1.17	5.69	±	1.29	0.611
Energy (kcal)	1586.91	±	432.68	1634.19	±	511.55	1606.97	±	462.76	0.073
Energy (kJ)	6639.63	±	1810.33	6837.45	±	2140.32	6723.56	±	1936.18	0.073
Carbohydrates (g)	221.43	±	72.68	228.94	±	59.63	65.47	±	224.62	0.352
Sugars (g)	86.09	±	42.2	92.2	±	32.6	88.8	±	38.3	0.092
Fats (g)	22.71	±	15.85	26.75	±	16.71	23.72	±	16.22	0.029
Cholesterol (mg)	174.09	±	134.93	199.95	±	122.31	127.82	±	180.67	0.041
Protein (g)	53.65	±	19.51	55.85	±	22.71	54.49	±	20.41	0.452
Fiber (g)	14.00	±	8.19	12.92	±	6.43	13.38	±	7.23	0.343

Normal body fat, <25% for girls and <30% for boys; *P* value comes from *t*-test.

Abbreviations: DED_fb_, dietary energy density food and beverage; DED_fo_, dietary energy density food only.

The children with high body fat had a significantly higher DED_fo_ (1.95 kcal/g, SD = 0.36) than children with normal body fat percent (1.85 kcal/g, SD = 0.32) (*P* = 0.011). Children with high body fat reported greater consumption of all macronutrients, although statistically significant differences were observed only in the case of fats (36.7 g, SD = 15.85 for children with normal body fat and 26.75 g, SD = 16.71 for children with high body fat; *P* = 0.029) and cholesterol (36.7 g, SD = 15.85 for children with normal body fat and 26.75 g, SD = 16.71 for children with high body fat; *P* = 0.041). Specifically, they reported a high intake of sugar sweetened drinks (for example, soda, “*atole*,” and juice drinks), “*tamales*,” fried snacks “*churros*,” and a high content of oil and lard in cooking (Supplemental Table 1).

In the adjusted linear regression models, DED_fo_ and DED_fb_ were not associated with any of the measurements of obesity (Table 3). However, both DED_fo_ and DED_fb_ were significantly associated with higher cholesterol concentrations [*β* = 11.67, 95% confidence interval (CI): 1.81, 19.53, *P* = 0.01; and *β* = 11.74, 95% CI: 2.69, 20.74, *P* = 0.01, respectively]. Neither DED_fo_ nor DED_fb_ were associated with BMIz, waist circumference, abdominal, or body fat percent.

**TABLE 3:** Linear regression model between the measures of adiposity and metabolic markers and DED in the studied children (*n* = 284)

	DED_fo_ (kcal/g)			DED_fb_ (kcal/g)		
	*β*	95% CI	*P*	*β*	95% CI	*P*
Waist circumference (cm)	0.69	(–0.7, 2.09)	0.62	1.3	(–0.3, 2.9)	0.42
BMI-for-age (*z*-score)	0.07	(–0.16, 0.30)	0.76	0.35	(–0.09, 0.62)	0.18
Body fat (%)	0.87	(–0.35, 2.1)	0.48	1.51	(–0.11, 2.92)	0.28
Abdominal fat (%)	1.05	(–0.65, 2.74)	0.54	1.96	(–0.01, 3.91)	0.32
Triglycerides (mg/dL)	3.45	(–9.17, 16.07)	0.59	3.46	(–11.16, 18.08)	0.64
Total cholesterol (mg/dL)	11.67	(1.81, 19.53)	0.03	11.74	(2.69, 20.79)	0.01
HDL (mg/dL)	1.51	(–1.26, 4.25)	0.28	0.23	(–2.97, 3.43)	0.88
LDL (mg/dL)	5.11	(–1.08, 11.3)	0.11	6.79	(–0.37, 13.95)	0.06

Model adjusted for age, sex, caretaker’s education level, and physical activity.

Abbreviations: *β*, beta coefficient from the regression model; CI, confidence interval; DED_fb_, dietary energy density food and beverage; DED_fo_, dietary energy density food only.

The adjusted logistic regression model in Table 4 shows that a higher DED_fo_ was strongly associated with having insulin resistance [odds ratio (OR) = 3.92, 95% CI: 1.66, 9.22, *P* < 0.01] and with higher odds of having higher concentrations of leptin (OR = 3.17, 95% CI: 1.01, 10.23, *P* = 0.04) and insulin (OR = 2.83, 95% CI: 1.28, 4.51, *P* = 0.01) (Table 3). Similarly, DED_fb_ was associated with insulin resistance (OR = 3.51, 95% CI: 1.25, 9.87, *P* = 0.02) and higher odds of having higher insulin concentration (OR = 2.95, 95% CI: 1.30, 6.70, *P* = 0.01) but was not associated with leptin concentrations. No association was found between DED_fo_ or DED_fb_ and the other markers of inflammation.

**TABLE 4:** Logistic regression model between HOMA, leptin, insulin, and inflammation markers with diet energy density (*n* = 284)

	OR	(95% CI)	*P*
DED_fo_ (kcal/g)			
HOMA (>3.16)	3.92	(1.66, 9.22)	<0.00
Leptin (>0.36 mg/L)	3.17	(1.01, 5.23)	0.04
Insulin (>10U/mL)	2.53	(1.26, 5.08)	0.01
IL-6 (>1.8 pg/mL)	2.06	(1.00, 4.21)	0.05
TNF-α (>3.5 pg/mL)	0.91	(0.47, 1.78)	0.79
CRP^1^ (>2.99 mg/L)	1.14	(0.84,1.32)	0.18
DED_fb_ (kcal/g)			
HOMA (>3.16)	3.51	(1.25, 9.87)	0.02
Leptin (>0.36 mg/L)	3.48	(0.62, 2.94)	0.07
Insulin (>10 U/mL)	2.95	(1.30, 6.70)	0.01
IL-6 (>1.8 pg/mL)	1.76	(0.81, 3.86)	0.16
TNF-α (>3.5 pg/mL)	0.64	(0.29, 1.40)	0.26
CRP^1^ (>0.99 mg/L)	1.86	(0.76, 4.52)	0.13

Adjusted model for body fat percent, age, sex, caretaker’s education level, physical activity, and C-reactive protein.

Abbreviations: CRP, C-reactive protein; DED_fb_, dietary energy density food and beverage; DED_fo_, dietary energy density food only; OR, odds ratio.

1 In this model, CRP was used as the independent variable; the median was used as the cutoff values for leptin, insulin, IL-6, and TNF-α; for CRP >2.99 mg/L, and HOMA >3.16.

## Discussion

This study assessed the DED of children from rural Mexico by 2 different methods “food only” and “food and beverage.” DED_fo_ and DED_fb_ were associated with higher cholesterol concentrations and higher odds of having higher leptin concentrations and insulin resistance. DED_fo_ was associated with percentage of body fat in the crude model; however, no association between DED_fo_ or DED_fb_ and any of the measures of obesity was found in the adjusted models.

The DED_fo_ of the children in this study was 1.91 kcal/g (SD = 0.36) and DED_fb_ was 1.36 kcal/g (SD = 0.31). The DED_fo_ from this study is similar to the DED_fo_ found in other pediatric populations. For instance, DED_fo_ IQR in children (5–13 y) in the United States was 2.08 (SD = 0.47) kcal/g [31] and an average of 1.64 kcal/g (SD = 0.23) was observed in children (5–13 y) in Germany [20]. In contrast, the DED_fo_ found in this study, in the United States and Germany, were higher than the one found in Japanese children of a similar age range (a DED_fo_ of 1.2 kcal/g; SD = 0.14) [32]. These differences might be explained by the difference in fat intake in each country. The diet of Japanese children tend to include more rice, seaweed, and vegetables, whereas diets in the United States tend to include more processed and energy-dense foods such as sweets, crisps, and fried foods [33] Furthermore, diets in EU and Germany include more meat and dairy products that are known to increase the DED [32,[34], [35], [36], [37]]. The higher DED found in the rural population studied in Mexico is related to the high intake of energy-dense foods (“*tamales*,” “*churros*”), the high content of oil and lard used to cook, the intake of highly processed foods, and the high intake of maize-based drinks in DED_fb_ [16], all commonly consumed in this population and reported to be consumed by the children in the study.

In this study, DED_fo_ was associated with percentage of body fat in the crude model; however, no association was observed between obesity and DED_fo_ or DED_fb_ in the adjusted model. Like our results, higher DED_fo_ or DED_fb_ was not associated with overweight or obesity, but was associated with stunting, in children living in an urban area in Malaysia [38]. In contrast, a study using representative data for the Mexican population, including urban and rural regions, found a positive association between DED and obesity in children [39]. Also, higher DED was associated with obesity in 2 studies in Spanish children [40]. However, one of these studies reported an association between DED DED_fo_ or DED_fb_ with body composition or cardiovascular disease risk factors only when excluding under-reporters. Our results excluded those reporting <3 SD of DED_fo_ or DED_fb_; thus, underreporting does not explain the lack of association that was observed. We need to consider the possibility that, given the high SD on both DED_fo_ and DED_fb_ observed in this population, a higher sample size might be required to determine if DED is associated with adiposity, because studies in Mexico with a higher sample size (*n* = 2600) have found an association between DED and obesity [39]. In addition, the fact that the significance level increased when including covariables indicates that socioeconomic factors may have a stronger influence on children’s body composition than DED does. Thus, the possibility that DED is associated with obesity in this population should not be discarded. Because of the lack of consistency observed in the literature, the relationship between DED and obesity in children should be explored further.

Both DED_fo_ and DED_fb_ were associated with higher cholesterol and DED_fo_ was associated with leptin concentrations. To the best of our knowledge, there are no studies assessing the relationship between leptin and cholesterol with DED. However, there are biological mechanisms that could explain these associations. In the case of leptin, it has been shown that dietary fat is associated with higher leptin concentration, independently from adiposity in children and adults [41,42]. In addition, leptin and leptin resistance have shown to influence food choices and increase food intake [43]. The association between DED_fo_ or DED_fb_ and higher concentrations of cholesterol might be attributable to dietary fat, which was higher in children with high body fat content. In contrast to the lack of association found between both DED_fo_ and DED_fb_ with measures of body composition after adjusting for covariables, the association between inflammation markers and lipids remained after controlling for confounders, indicating that DED could have an influence on the children’s metabolism independently of socioeconomic factors.

This is the first study to observe and association between DED_fo_ and DED_fb_ and higher odds of insulin resistance in children. In adults from Europe and United States, DED was associated with diabetes and insulin resistance [7,44]. Furthermore, similar to our results, Mendoza et al. [45] found that the association between DED and insulin resistance was independent from adiposity in adults from the United States. One of the metabolic mechanism that might explain this association is the high content of fat and sugar, which were commonly consumed by the children of this study and other studies [33,46]. Dietary fats and sugars are known to increase systemic inflammation [47] and may contribute to the higher levels of insulin and insulin resistance observed in this population.

In contrast with other studies, where conflicting results were found between the different DED calculation methods (food only compared with food and beverage), we found no substantial differences in the strength and the direction of the association between the different methods. For instance, a study by McCaffrey et al. [48] found that DED_fb_ was not associated with body fat, whereas DED_fo_ was associated with a higher body fat in children and adolescents [49]. As reported by other studies evaluating the use of different DED methods, in this study, the variation within subject of the DED_fo_ was lower than DED_fb_ [10].

Understanding the DED in this population is important for addressing potential nutritional challenges and promoting overall health. Identifying patterns of energy-dense food consumption can help develop targeted interventions and nutritional education programs to enhance health and well-being in this population. This study provides valuable insights into the complex interplay of dietary habits with chronic conditions while taking in consideration cultural, socioeconomic, and environmental factors. This research may contribute to the broader understanding of nutritional patterns in diverse populations and aid in the development of evidence-based strategies for improving children’s health in rural settings [50].

One limitation of the study is that causality cannot be inferred between DED, obesity, and chronic disease markers because of the cross-sectional design. Another limitation relates to systematic underreporting in dietary intake data [51]. As mentioned before, given the high SD of both DED evaluated, a higher sample size might be needed to evaluate the association between DED and adiposity. It is important to consider that other possible confounders that may influence the association between DED, obesity, and biomarkers of inflammation, such as growth spurs, genetic predisposition, as well as bacterial infections, among others, were not measured and, thus, were not controlled for in the statistical analysis. Despite these limitations, our study is the first to assess the relationship between DED, inflammatory markers, and insulin resistance, providing information on the implications of a high DED in children living in rural areas beyond obesity.

In conclusion, both, DED_fo_ and DED_fb_, were not associated with measurements of obesity in children from rural Mexico, but they were associated with higher cholesterol and insulin resistance, whereas DED_fb_ was related to higher leptin concentrations. Reducing DED may result in a lower risk of cardiovascular disease, better insulin sensitivity, and inflammatory profile in children living in rural Mexico.

## Author contributions

The authors’ responsibilities were as follows – GAZ, OPGO: conceived the study; DR: collected the data; JLR, OPGO: provided important contextual information; MC: performed the laboratory work; GAZ, OPGO, MCC: developed the analysis plan; GAZ, MCC: revised the data and conducted the statistical analysis; GAZ, OPGO, JLR: wrote the manuscript; and all authors: revised and approved the manuscript.

### Conflict of interest

GAZ reports financial support, administrative support, and article publishing charges were provided by University of York. Gerardo Zavala reports a relationship with University of York. All other authors report no conflicts of interest.

### Funding

This study was partially funded by Consejo Nacional de Ciencia y Tecnología (CONACyT) México, which provided the PhD grant 218666. Article publishing charges were provided by University of York.

### Data availability

The data are available upon request to the corresponding author.

## Appendix A Supplementary data

Multimedia component1 Multimedia component1

## References

[1] Shamah-Levy T., Gaona-Pineda E.B., Cuevas-Nasu L., Morales-Ruan C., Valenzuela-Bravo D.G., Humaran I.M. (2023). Prevalencias de sobrepeso y obesidad en población escolar y adolescente de México. Ensanut Continua 2020–2022. Salud Pública Méx..

[2] Ávila-Curiel A., Galindo-Gómez C., Juárez-Martínez L., Osorio-Victoria M.L. (2018). Síndrome metabólico en niños de 6 a 12 años con obesidad, en escuelas públicas de siete municipios del Estado de México. Salud Pública Méx..

[3] Noubiap J.J., Nansseu J.R., Lontchi-Yimagou E., Nkeck J.R., Nyaga U.F., Ngouo A.T. (2022). Global, regional, and country estimates of metabolic syndrome burden in children and adolescents in 2020: a systematic review and modelling analysis. Lancet Child Adolesc. Health..

[4] López Rodríguez G., Galván M., Fuentes Galicia S.J. (2016). Indicadores de síndrome metabólico en escolares mexicanos con talla baja, sobrepeso u obesidad. Arch. Latinoam. Nutr..

[5] Bell E.A., Castellanos V.H., Pelkman C.L., Thorwart M.L., Rolls B.J. (1998). Energy density of foods affects energy intake in normal-weight women. Am. J. Clin. Nutr..

[6] World Health Organization (2003).

[7] Vernarelli J.A., Mitchell D.C., Rolls B.J., Hartman T.J. (2015). Dietary energy density is associated with obesity and other biomarkers of chronic disease in US adults. Eur. J. Nutr..

[8] Donin A.S., Nightingale C.M., Owen C.G., Rudnicka A.R., Jebb S.A., Ambrosini G.L. (2014). Dietary energy intake is associated with type 2 diabetes risk markers in children. Diabetes Care.

[9] Ledikwe J.H., Rolls B.J., Smiciklas-Wright H., Mitchell D.C., Ard J.D., Champagne C. (2007). Reductions in dietary energy density are associated with weight loss in overweight and obese participants in the PREMIER trial. Am. J. Clin. Nutr..

[10] Perez-Escamilla R., Obbagy J.E., Altman J.M., Essery E.V., McGrane M.M., Wong Y.P. (2012). Dietary energy density and body weight in adults and children: a systematic review. J. Acad. Nutr. Diet..

[11] Vernarelli J.A., Mitchell D.C., Hartman T.J., Rolls B.J. (2011). Dietary energy density is associated with body weight status and vegetable intake in U.S. children. J. Nutr..

[12] Johnson L., Mander A.P., Jones L.R., Emmett P.M., Jebb S.A. (2008). A prospective analysis of dietary energy density at age 5 and 7 years and fatness at 9 years among UK children. Int. J. Obes. (Lond)..

[13] Mendoza J.A., Drewnowski A., Cheadle A., Christakis D.A. (2006). Dietary energy density is associated with selected predictors of obesity in U.S. children. J. Nutr..

[14] Butte N.F., Cai G., Cole S.A., Wilson T.A., Fisher J.O., Zakeri I.F. (2007). Metabolic and behavioral predictors of weight gain in Hispanic children: the Viva la Familia Study. Am. J. Clin. Nutr..

[15] Moreno-Altamirano L., Hernandez-Montoya D., Silberman M., Capraro S., Garcia-Garcia J.J., Soto-Estrada G. (2014). [The nutrition transition and the double burden of malnutrition: changes in dietary patterns 1961–2009 in the Mexican socioeconomic context]. Arch. Latinoam. Nutr..

[16] González-Castell D., González-Cossío T., Barquera S., Rivera J.A. (2007). Alimentos industrializados en la dieta de los preescolares mexicanos. Salud Publica Mex.

[17] Ledikwe J.H., Blanck H.M., Khan L.K., Serdula M.K., Seymour J.D., Tohill B.C. (2005). Dietary energy density determined by eight calculation methods in a nationally representative United States population. J. Nutr..

[18] Romero-Polvo A., Denova-Gutiérrez E., Rivera-Paredez B., Castañón S., Gallegos-Carrillo K., Halley-Castillo E. (2012). Association between dietary patterns and insulin resistance in Mexican children and adolescents. Ann. Nutr. Metab..

[19] Zavala G.A., Garcia O.P., Campos-Ponce M., Ronquillo D., Caamano M.C., Doak C.M. (2016). Children with moderate-high infection with *Entamoeba coli* have higher percentage of body and abdominal fat than non-infected children. Pediatr. Obes..

[20] Gunther A.L., Stahl L.J., Buyken A.E., Kroke A. (2011). Association of dietary energy density in childhood with age and body fatness at the onset of the pubertal growth spurt. Br. J. Nutr..

[21] Van der Aa M.P., Fazeli Farsani S., Knibbe C.A., de Boer A., van der Vorst M.M. (2015). Population-based studies on the epidemiology of insulin resistance in children. J. Diabetes Res..

[22] Lohman T., Roche A., Martorell R. (1988).

[23] de Onis M, Onyango A.W., Borghi E., Siyam A., Nishida C., Siekmann J. (2007). Development of a WHO growth reference for school-aged children and adolescents. Bull. World Health Organ..

[24] Hill A.M., LaForgia J., Coates A.M., Buckley J.D., Howe P.R. (2007). Estimating abdominal adipose tissue with DXA and anthropometry. Obesity (Silver Spring).

[25] Ellis K.J., Abrams S.A., Wong W.W. (1997). Body composition of a young, multiethnic female population. Am. J. Clin. Nutr..

[26] Chavez M., Chavez A., Roldan J., Pérez-Gil S., Hernández S. (1996).

[27] Cox D.N., Mela D.J. (2000). Determination of energy density of freely selected diets: methodological issues and implications. Int. J. Obes. Relat. Metab. Disord..

[28] Hernández B., Gortmaker S.L., Laird N.M., Colditz G.A., Parra-Cabrera S., Peterson K.E. (2000). Validez y reproducibilidad de un cuestionario de actividad e inactividad física para escolares de la ciudad de México. Salud Publ. Mex..

[29] Ainsworth B.E., Haskell W.L., Whitt M.C., Irwin M.L., Swartz A.M., Strath S.J. (2000). Compendium of physical activities: an update of activity codes and MET intensities. Med. Sci. Sports Exerc..

[30] Harmse B., Kruger H.S. (2010). Significant differences between serum CRP levels in children in different categories of physical activity: the PLAY study: cardiovascular topics. Cardiovasc. J. Afr..

[31] Cedillo Y.E., Garr-Barry V., Maciel B., Fernández J.R (2019). Dietary energy–density and adiposity markers among a cohort of multi-ethnic children. Matern. Child Health J..

[32] Murakami K., Miyake Y., Sasaki S., Tanaka K., Arakawa M. (2012). An energy-dense diet is cross-sectionally associated with an increased risk of overweight in male children, but not in female children, male adolescents, or female adolescents in Japan: the Ryukyus Child Health Study. Nutr. Res..

[33] Alonzo S.A.S., Jiménez D.J.J., Zavala H.A. (2018). Patrones de consumo de alimentos ultra procesados, dieta y caracteristicas somatometricas en cinco niños de edad escolar. Jóvenes En La Ciencia..

[34] Micha R., Khatibzadeh S., Shi P., Fahimi S., Lim S., Andrews K.G. (2014). Global, regional, and national consumption levels of dietary fats and oils in 1990 and 2010: a systematic analysis including 266 country-specific nutrition surveys. BMJ.

[35] Qi Q., Chu A.Y., Kang J.H., Huang J., Rose L.M., Jensen M.K. (2014). Fried food consumption, genetic risk, and body mass index: gene-diet interaction analysis in three US cohort studies. BMJ.

[36] De Lira-García C., Bacardí-Gascón M., Jiménez-Cruz A. (2012). Preferences of healthy and unhealthy foods among 3 to 4 year old children in Mexico, Asia Pac. J. Clin. Nutr..

[37] Bel-Serrat S., Mouratidou T., Börnhorst C., Peplies J., De Henauw S., Marild S. (2013). Food consumption and cardiovascular risk factors in European children: the IDEFICS study. Pediatr. Obes..

[38] Shariff Z.M., Lin K.G., Sariman S., Siew C.Y., Yusof B.N.M., Mun C.Y. (2016). Higher dietary energy density is associated with stunting but not overweight and obesity in a sample of urban Malaysian children. Ecol. Food Nutr..

[39] Aburto T.C., Cantoral A., Hernández-Barrera L., Carriquiry A.L., Rivera J.A. (2015). Usual dietary energy density distribution is positively associated with excess body weight in Mexican children. J. Nutr..

[40] Gomez-Bruton A., Arenaza L., Medrano M., Mora-Gonzalez J., Cadenas-Sanchez C., Migueles J.H. (2019). Associations of dietary energy density with body composition and cardiometabolic risk in children with overweight and obesity: role of energy density calculations, under-reporting energy intake and physical activity. Br. J. Nutr..

[41] Chu N.E., Stampfer M.J., Spiegelman D., Rifai N., Hotamisligil G.S., Rimm E.B. (2001). Dietary and lifestyle factors in relation to plasma leptin concentrations among normal weight and overweight men. Int. J. Obes. Relat. Metab. Disord..

[42] Klok M.D., Jakobsdottir S., Drent M.L. (2007). The role of leptin and ghrelin in the regulation of food intake and body weight in humans: a review. Obes. Rev..

[43] Vasselli J.R. (2012). The role of dietary components in leptin resistance. Advances in nutrition.

[44] Wang J., Luben R., Khaw K.T., Bingham S., Wareham N.J., Forouhi N.G. (2008). Dietary energy density predicts the risk of incident type 2 diabetes: the European Prospective Investigation of Cancer (EPIC)-Norfolk Study. Diabetes Care.

[45] Mendoza J.A., Drewnowski A., Christakis D.A. (2007). Dietary energy density is associated with obesity and the metabolic syndrome in U.S. adults. Diabetes Care.

[46] Rolls B.J. (2000). The role of energy density in the overconsumption of fat. J. Nutr..

[47] Zavala G., Long K.Z., García O.P., del Carmen Caamaño M., Aguilar T., Salgado L.M. (2013). Specific micronutrient concentrations are associated with inflammatory cytokines in a rural population of Mexican women with a high prevalence of obesity. Br. J. Nutr..

[48] McCaffrey T.A., Rennie K.L., Kerr M.A., Wallace J.M., Hannon-Fletcher M.P., Coward W.A. (2008). Energy density of the diet and change in body fatness from childhood to adolescence; is there a relation?. Am. J. Clin. Nutr..

[49] Vergnaud A.C., Estaquio C., Czernichow S., Péneau S., Hercberg S., Galan P. (2009). Energy density and 6-year anthropometric changes in a middle-aged adult cohort. Br. J. Nutr..

[50] Drewnowski A., Dwyer J., King J.C., Weaver C.M. (2019). A proposed nutrient density score that includes food groups and nutrients to better align with dietary guidance. Nutr. Rev..

[51] Amanatidis S., Mackerras D., Simpson J.M. (2001). Comparison of two frequency questionnaires for quantifying fruit and vegetable intake. Public Health Nutr.

