# Supplementary material for: Dietary Energy Density Is Associated with Biomarkers of Chronic Diseases—A Cross-Sectional Study of School-Aged Children in Rural Mexico
Source: Curr Dev Nutr. 2024 Feb 17;8(3):102096. doi: 10.1016/j.cdnut.2024.102096 (PMC10924138; doi:10.1016/j.cdnut.2024.102096)
Supplement: Multimedia component1 [file mmc1.docx]

**Dietary energy density is associated with biomarkers of chronic diseases - A cross-sectional study of school-aged children in rural Mexico**

Gerardo A Zavala

**Supplementary table 1.** Food groups and items consumed by the of the studied children according to their body fat content.

|  | Normal body fat (n=131)  Mean ± SD | | | High body fat (n=153)  Mean ± SD | | | Overall (n=284)  Mean ± SD | | | *p* |
| --- | --- | --- | --- | --- | --- | --- | --- | --- | --- | --- |
| Fruits (g per day) | 204.69 | ± | 146.80 | 205.50 | ± | 178.08 | 205.03 | ± | 160.51 | 0.154 |
| Vegetables (g per day) | 67.47 | ± | 72.26 | 58.14 | ± | 67.22 | 62.09 | ± | 69.43 | 0.854 |
| Cereals (g per day) | 276.41 | ± | 112.75 | 285.13 | ± | 121.57 | 280.11 | ± | 116.46 | 0.201 |
| Legumes (g per day) | 78.75 | ± | 92.26 | 68.22 | ± | 66.61 | 72.68 | ± | 78.53 | 0.523 |
| Dairy (g per day) | 244.77 | ± | 165.09 | 254.05 | ± | 138.43 | 250.12 | ± | 149.98 | 0.421 |
| Meat (g per day) | 132.61 | ± | 84.82 | 135.53 | ± | 86.82 | 134.29 | ± | 85.85 | 0.205 |
| Oil (g per day) | 34.55 | ± | 78.07 | 38.89 | ± | 80.03 | 37.04 | ± | 79.08 | 0.089 |
| Sugar sweetened beverages (ml per day) | 27.25 | ± | 3.47 | 33.89 | ± | 4.87 | 29.00 | ± | 6.79 | 0.041 |
| *Atole* (ml per day) | 33.10 | ± | 38.25 | 48.25 | ± | 49.30 | 40.23 | ± | 45.23 | 0.077 |
| Box cereal (g per day) | 6.84 | ± | 15.67 | 7.25 | ± | 13.67 | 7.08 | ± | 14.53 | 0.881 |
| Fried *churros* (g per day) | 12.30 | ± | 16.03 | 14.66 | ± | 20.08 | 16.56 | ± | 22.52 | 0.524 |
| Desserts and pastries (g per day) | 8.21 | ± | 17.19 | 9.29 | ± | 25.87 | 9.05 | ± | 25.87 | 0.092 |
| Milkshakes (flavored milk with sugar) (g per day) | 28.57 | ± | 57.41 | 34.57 | ± | 54.42 | 30.21 | ± | 56.55 | 0.084 |
| Red meat (g per day) | 51.10 | ± | 55.60 | 42.06 | ± | 44.96 | 45.89 | ± | 49.36 | 0.254 |
| *Atole*: maize-based sweetened hot beverage; *Churros:* wheat flour dough, fried in vegetable oil and covered in sugar | | | | | | | | | | |
|  | | | | | | | | | | |
